# Effects of Endic Anhydride Grafted PPC on the Properties of PHBV Blends

**DOI:** 10.3390/ma15176179

**Published:** 2022-09-05

**Authors:** Qing Zhang, Yongguang Gao, Huiyuan Liu, Shili Shu, Wei Chen

**Affiliations:** College of Chemistry, Tangshan Normal University, Tangshan 063000, China

**Keywords:** poly(β-hydroxybutyrate-*co*-β-hydroxyvalerate), poly(propylene carbonate), endic anhydride, graft modification

## Abstract

Poly(β-hydroxybutyrate-*co*-β-hydroxyvalerate) (PHBV) was modified with endic anhydride grafted poly(propylene carbonate) (EA–PPC), and then PHBV/EA–PPC composite polymers were prepared by melt blending under the catalysis of stannous octoate (Sn(Oct)_2_). The blends were characterized by an electronic universal testing machine, cantilever impact testing machine, and differential scanning calorimeter (DSC), as well as dynamic mechanical analysis (DMA) and field emission scanning electron microscopy (FESEM). Effects of the amount of Sn(Oct)_2_ on the mechanical properties, thermal properties, and morphology of the blends were discussed. The results showed that the addition of Sn(Oct)_2_ promoted the transesterification reaction between PHBV and EA–PPC, and the compatibility between PHBV and PPC was greatly improved. When the amount of Sn(Oct)_2_ was 3 wt%, the impact strength and elongation at break of the PHBV/EA–PPC blend increased from 3.7 kJ/m^2^ and 4.1% to 5.9 kJ/m^2^ and 387.5%, respectively, and there was no significant decrease in tensile strength. Additionally, four esterification reaction mechanisms for PHBV/EA–PPC blends were proposed.

## 1. Introduction

**Scheme 1:**
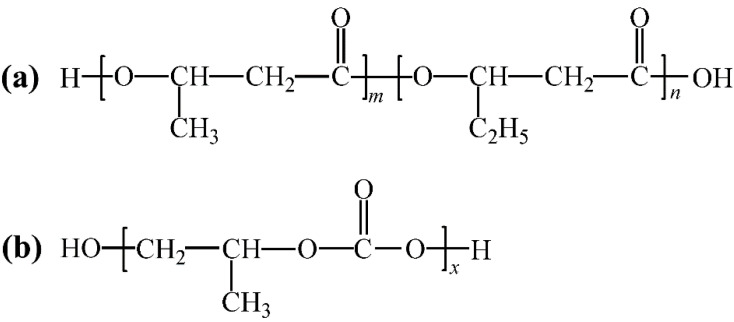
Structures of (**a**) PHBV and (**b**) PPC.

Among the polymer materials obtained by microbial fermentation, the most commercialized ones are currently polyhydroxyalkanoates (PHAs) [1,2,3]. PHAs are a family of linear biopolyesters composed of hydroxyalkanoate (HA) units, which are generally obtained by microorganisms under the condition of an imbalance of nutrients such as carbon and nitrogen, and can be stored in cells as energy and a carbon source [4,5,6]. Poly(β-hydroxybutyrate-*co*-β-hydroxyvalerate) (PHBV) is one member of the PHA family and its structure contains randomly arranged 3-hydroxybutyrate (HB) and 3-hydroxyvalerate (HV) segments. The structure is shown in Scheme 1a [4]. Because the segment structures of HB and HV are very similar, the phenomenon of isodimorphism can be seen during the crystallization of PHBV, resulting in a high crystallinity. Moreover, there is a serious post-crystallization phenomenon, which brings great difficulty to the processing [7,8]. PHBV exhibits excellent biocompatibility, optical activity, piezoelectricity, gas barrier properties, etc. [9,10,11,12]. It has been widely used in many fields such as packaging materials, tissue engineering materials, and sustained-release materials. However, the low glass transition temperature of PHBV causes its secondary crystallization during storage at room temperature, resulting in increased brittleness [13,14]. The low impact resistance and poor processability, resulting from its very high crystallinity, delay its commercial applications on a large scale [10,15,16,17]. Therefore, PHBV must be modified to broaden its applications. A series of studies have been performed to enhance its flexibility and mechanical properties, where PHBV was blended with various fillers [18,19,20] or compounded with various thermoplastic polymers [21,22,23].

Polypropylene carbonate (PPC) is an environmentally friendly and biodegradable aliphatic polycarbonate obtained by alternating copolymerization of carbon dioxide and propylene oxide. The production process can consume a large amount of the greenhouse gas carbon dioxidec [24,25]. Its structure is shown in Scheme 1b. There are ether bonds on the main chain of PPC, so the molecular chain is easy to rotate around the ether bond, which makes PPC very flexible with excellent tensile toughness, very high elongation at break, but poor strength [26,27]. Based on the complementarity of the mechanical properties of PHBV and PPC, melt blending PPC with PHBV will not only enhance the toughness of PHBV but also modify the low strength and bad thermal stability of PPC. However, the interfacial compatibility of PHBV and PPC is poor [28], and simple blending cannot effectively improve the mechanical properties of composites, so they need to be compatibilized.

In the present work, endic anhydride grafted poly(propylene carbonate) (EA–PPC) was prepared by grafting PPC with endic anhydride (EA), and the EA–PPC acted as an in situ interfacial compatibilizer. Stannous octoate (Sn(Oct)_2_) is a widely used catalyst and had been proved to be more efficient than some other catalysts for catalyzing the transesterification between PLA and other polyesters [29,30,31]. PHBV/EA–PPC blends were prepared by melt transesterification under the catalysis of Sn(Oct)_2_, and their properties were characterized. Effects of the amount of catalyst on the thermal properties, mechanical properties, and morphology of the blends were discussed.

## 2. Materials and Methods

### 2.1. Materials

PHBV (Y1000P) and PPC (PPC101) were commercially obtained from Tianan Biomaterials Co., Ltd. (Ningbo, China) and Nanyang Zhongju Tianguan Low Carbon Technology Co., Ltd. (Nanyang, China), respectively. Endic anhydride (an endo isomer) with a full name of bicyclo [2.2.1]hept-5-ene-2,3-dicarboxylic anhydride) (analytical reagent) (AR), Sn(Oct)_2_ (AR), chloroform (AR), potassium hydroxide (KOH) (AR), and phenolphthalein (AR) were purchased from Sinopharm Chemical Reagent Co, Ltd. (Shanghai, China). Polyethylene wax (PE wax) was supplied by Qingdao Sino New Material Co., Ltd. (Qingdao, China). All reagents were used without any further purification.

### 2.2. Preparation Procedures

#### 2.2.1. Preparation of EA–PPC

PPC was dried in a vacuum drying oven at 50 °C for 12 h before use. The dried PPC and EA were premixed in a high-speed disperser for 5 min at a mass ratio of 99:1 and then added to the mixing chamber of a torque rheometer to be melt-blended for 8 min at a temperature of 145 °C and a rotational speed of 40 rpm. After the torque value reached an equilibrium, we stopped the motor to obtain EA–PPC. The samples were pelletized after cooling and placed in a desiccator for use.

#### 2.2.2. Blend Preparation of PHBV Composites

PHBV, PPC, and PE wax were dried in a vacuum drying oven at 50 °C for 24 h before use. The dried raw materials were weighed and premixed in a high-speed mixer for 5 min according to the formulations listed in Table 1. The premixed blends were poured into the mixing chamber of the torque rheometer for 8 min under a temperature of 175 °C and a rotational speed of 40 rpm. After the torque value reached an equilibrium, the motor of the torque rheometer was stopped to obtain PHBV/PPC blends. Then, the resulting blends were pelletized after cooling. Next, the blend pellets were added to the barrel of a microinjection molder and injected into standard splines for testing. The temperature of the mold zone and the injection zone were set to 50 and 185 °C, respectively. The injection time and the holding time were 5 s and 40 s, respectively.

PHBV/EA–PPC blends were also prepared according to the above procedure. Here, the ratio of PHBV and EA–PPC was fixed at 70:30. The dried PHBV, EA–PPC, a certain amount of Sn(Oct)_2_, and 0.5 g PE wax was mixed in a high-speed disperser. The melting reaction was also carried out in the mixing chamber of the torque rheometer. Finally, the standard splines of the PHBV/EA–PPC blends were obtained for testing.

### 2.3. Analysis

The grafting degree (*D_g_*) of EA–PPC was determined by referring to the literature [32,33]. EA–PPC was dissolved in 5 wt/v% chloroform and then precipitated three times with excess ethanol to remove free EA. The purified EA–PPC was dried in a vacuum oven at 50 °C for 24 h and then placed in a desiccator for use. A total of 0.8 g of dried EA–PPC was dissolved in 100 mL of chloroform. The EA–PPC/chloroform solution was titrated with 0.01 mol L^−1^ KOH/ethanol solution with phenolphthalein as an indicator. A blank experiment for neat PPC was performed according to the same procedure. The *D_g_* was calculated with the following formula.

$$D_{g}\;\left(\%\right)=\frac{\left(V_{1}-V_{0}\right)\times M_{EA}}{2\times 10^{-5}\times w}\times 100\%$$

where *V*_1_ and *V*_0_ (mL) are the volumes of KOH solution consumed by EA–PPC and PPC, respectively. *M_EA_* (g mol-1) and *w* (g) are the molecular weight of MA and the weight of EA–PPC, respectively.

Tensile properties were carried out using a CMT4202 universal testing machine (Shenzhen, China) according to ASTM D638, at a test speed of 10 mm/min. Specimens were dumbbell-shaped with dimensions of 75 mm × 4 mm × 1 mm. Izod impact properties of notched samples were determined with an XJU-22 impact tester (Suzhou, China) according to ASTM D-256 standard.

TGA analyses were performed with a PerkinElmer TGA 4000 thermogravimetric analyzer (Waltham, MA, USA) at a heating rate of 10 °C/min from 20 to 550 °C under a nitrogen atmosphere.

DSC measurements were carried out on a PerkinElmer DSC 4000 instrument (Waltham, MA, USA) under a nitrogen atmosphere. The heating and cooling rates were all set to 10 °C/min. The crystallinity degree (*χ*_*c*_) was calculated through the following formula [21]:

$$\chi_{c}\left(\%\right)=\frac{\Delta H_{m}}{\Delta H_{m}^{0}\times \omega_{PHBV}}\times 100\%$$

where $\Delta H_{m}$ is the melting enthalpy of the PHBV blend, $\Delta H_{m}^{0}$ is the melting enthalpy of 100% crystalline for PHBV (146 J/g) [34], and $\omega_{PHBV}$ is the weight fraction of PHBV in the sample. $\Delta H_{m}$ was derived from the second heating DSC curve.

DMA analyses were determined by a DMA Q800 TA Instruments (New Castle, DE, USA) in single cantilever mode on rectangular samples with the dimension of 40 mm × 10 mm × 4 mm. The test frequency was set at 1 Hz, and the samples were heated from −30 to 80 °C at a heating rate of 2 °C/min.

The morphology of the fractured surfaces of specimens was observed with a ZEISS Sigma 300 FESEM (Schnelldorf, Germany) with an accelerating voltage of 10 kV. The samples were sprayed with gold.

## 3. Results

### 3.1. Mechanical Properties of PHBV Blends

**Scheme 2:**
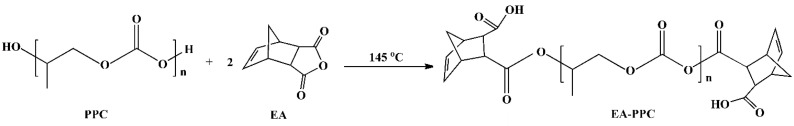
End-capping of PPC with EA.

**Figure 1:**
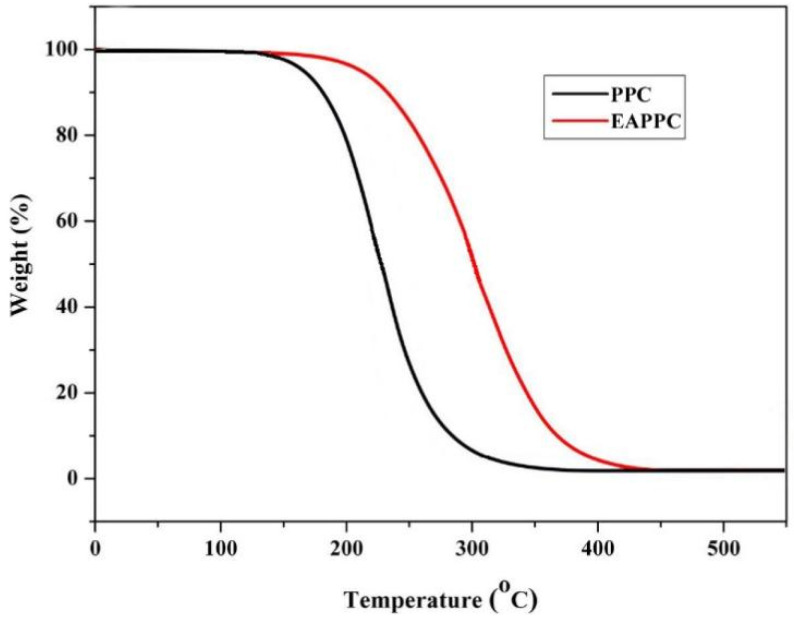
TGA curves of PPC and EA–PPC.

Table 1 presents the mechanical property data of PHBV, PPC, and PHBV/PPC blends with or without the transesterification catalyst Sn(Oct)_2_. Neat PHBV exhibited high rigidity with a tensile strength of 39.6 MPa, but poor impact strength and low elongation at break at the values only of 3.7 kJ/m^2^ and 4.1%, respectively, indicating it was a relatively stiff and brittle polymer. On the other hand, PPC was a ductile polymer with up to about 483.7% of elongation at break but a low tensile strength of 13.8 MPa. The tensile strength, impact strength, and elongation at break of the PLA/PPC blends were also low when PHBV and PPC were blended directly without the catalyst in different proportions. Even after adding Sn(Oct)_2_, the toughness of blends was still not improved. This was because the ungrafted PPC underwent extensive thermal degradation and chain scission during the melt processing at 175 °C, which was a drag on the mechanical properties of PHBV/PPC blends. In such a case, the terminal hydroxyl groups of PPC were capped by grafting the EA moieties, and then the zipper-type thermal degradation was effectively inhibited. The route for end-capping of PPC with EA is shown in Scheme 2. EA–PPC was obtained by the alcoholysis and acidolysis reaction of hydroxyl and carboxyl groups at both ends of the PPC chain with an equivalent amount of EA, respectively. The *Dg* of EA on the PPC chain was 0.26%, which was obtained from the titration test. The improved thermal stability of EA–PPC is demonstrated by thermogravimetric analysis (TGA) in Figure 1. From Figure 1 we can see that the onset decomposition temperature (the temperature at 5% weight loss) of EA–PPC shifted to a higher value (213.6 °C) compared with that of neat PPC (162.5 °C).

**Figure 2:**
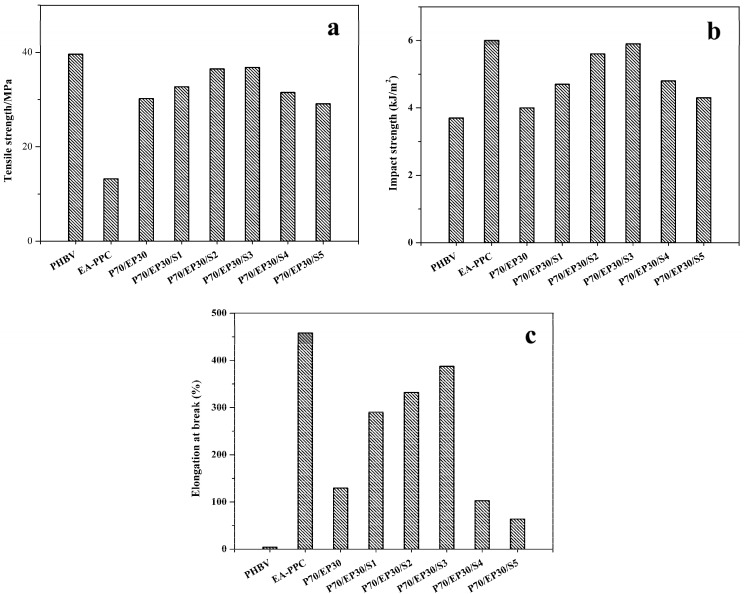
Mechanical properties of PHBV/EA–PPC blends: (**a**) tensile strength, (**b**) impact strength, and (**c**) elongation at break.

Sn(Oct)_2_ was used as a catalyst for the transesterification of PHBV and EA–PPC, and the effect of Sn(Oct)_2_ dosage on the mechanical properties of PHBV/EA–PPC blends was investigated. Here, PHBV was blended with EA–PPC in a weight ratio of 70/30, and the amount of catalyst was varied from 0 to 5 parts (wt%). Figure 2 shows the mechanical properties of PHBV/EA–PPC blends with different amounts of the catalyst, and the relevant data are summarized in Table 2.

Due to the toughness of PPC, the elongation at break of EA–PPC was also as high as 458.1%, while the tensile strength was only 13.2 MPa. Compared with neat PHBV, although the tensile strength of the PHBV/EA–PPC blends decreased, the impact strength and elongation at break were greatly improved. With the increase of Sn(Oct)_2_ content, the tensile strength, impact strength, and elongation at break of the blends all showed a trend of increasing first and then decreasing. As shown in Figure 2a and Table 2, when the Sn(Oct)_2_ content was increased to 3 parts, the tensile strength of the blends increased to 36.8 MPa, which was not significantly reduced compared with neat PHBV. With 3 parts of the catalyst, the impact strength and elongation at break reached 5.9 kJ/m^2^ and 387.5%, respectively (Figure 2b,c). However, after the amount of Sn(Oct)_2_ exceeded 3 parts, the tensile strength, impact strength, and elongation at break of blends all decrease gradually, especially the elongation at break, which decreased obviously. This was because an appropriate amount of catalyst could effectively catalyze the transesterification reaction between PHBV and EA–PPC, which increased the interfacial compatibility and enhanced the interaction between molecular chains at the interface of the two polymers so that the external stresses can be absorbed and transferred effectively between the polymers. Although the addition of excess catalysts increased the degree of transesterification, the scission of the macromolecular chains also occurred at the same time. So, quantities of PHBV and PPC oligomers were generated during the blending process, resulting in a decrease in the mechanical properties of the blends.

### 3.2. Thermal Properties of PHBV/EA–PPC Blends

**Figure 3:**
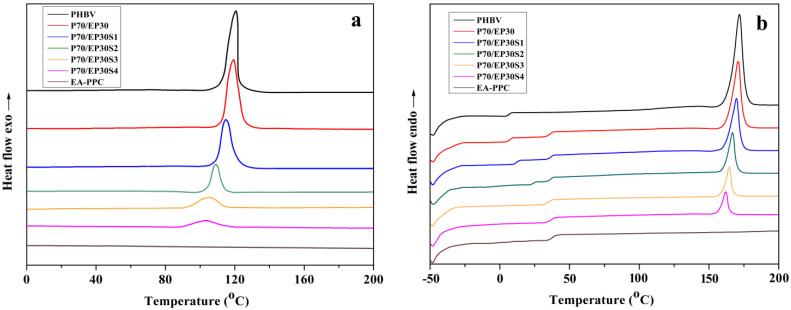
DSC curves of neat PHBV, EA–PPC, and PHBV/EA–PPC blends with various amounts of Sn(Oct)_2_: (**a**) cooling, (**b**) the second heating.

Figure 3a,b show the DSC cooling curves and secondary heating curves of the PHBV/PPC blends at a cooling rate of 10 °C/min and a heating rate of 10 °C/min, respectively. The effect of Sn(Oct)_2_ content was investigated and the relevant data are summarized in Table 3.

It can be seen that no crystallization peak appeared on the DSC cooling curve of EA–PPC, and there was also no melting peak, while only the glass transition process could be observed on the second heating curve. This was attributed to the fact that EA–PPC is an amorphous polymer. The crystallization temperature (*T_c_*) of neat PHBV was 120.6 °C, and only the crystallization peak of PHBV appeared in the cooling curves of PHBV/EA–PPC blends. With the increase in Sn(Oct)_2_ content, the crystallization temperature of PHBV/PPC blends shifted gradually to a lower value. When the amount of Sn(Oct)_2_ was 1, 2, 3, and 4 parts, compared with the neat PHBV, the crystallization temperature of blends decreased by 5.9, 11.4, 15.4, and 17.5 °C, respectively. The addition of Sn(Oct)_2_ enhanced the interaction between PHBV and EA–PPC, and more flexible EA–PPC chains were introduced into the PHBV molecules. On one hand, the flexible and amorphous EA–PPC in the blends acted as a “solvent” to “dilute” the nuclei of PHBV. On the other hand, it destroyed the stereoregularity of the PHBV molecular chain and then inhibited the crystallization of PHBV in the blends. In order to further verify the effect of Sn(Oct)_2_ on the crystallization ability of the blends, the crystallinity ($\chi_{c}$) of PHBV in the blends was calculated from the second heating curve of DSC (Figure 3b) and is listed in Table 3. One can see that the crystallinity of the blends decreased with the increase in the amount of Sn(Oct)_2_.

The glass transition temperature (*T_g_*) and melting endothermic peak temperature (*T_m_*) of neat PHBV were 6.3 and 171.7 °C, respectively. For the blend P70/EP30 without Sn(Oct)_2_ added, the DSC curve showed two distinctive *T_g_* values, which corresponded to the respective *T_g_* value of the two components, indicating that the compatibility of PHBV and EA–PPC was poor, and there was an obvious interface between them. As the amount of Sn(Oct)_2_ increased, the difference between the two *T_g_* values decreased gradually, until the amount reached 3 parts (P70/EP30/S3 blends). At this point, the *T_g_* values of two components merged into one, which represented that compatibility of the two components was improved via adding Sn(Oct)_2_ as a catalyst for the transesterification between PHBV and EA–PPC. For the PHBV blends, *T_g_* increased compared to neat PHBV, which solved the problem of the secondary crystallization of PHBV during storage at room temperature. The *T_m_* of the PHBV blends presented a decreasing trend with the increasing amount of Sn(Oct)_2_. This was because the low-temperature crystallization of polymer chains made the crystallization not perfect, and then the imperfect crystals will melt at lower temperatures. The lowering of *T_m_* was beneficial for the broadening of the processing window of the blends.

**Figure 4:**
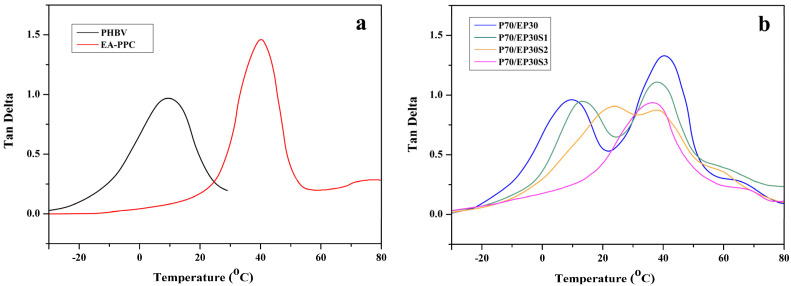
DMA of neat polymers (**a**) and PHBV/EA–PPC blends (**b**).

The glass transition of the blends could also be identified from the DMA thermograms. Figure 4 depicts the damping factor (tan δ) of neat PHBV, EA–PPC, and PHBV/EA–PPC blends with various amounts of Sn(Oct)_2_ as a function of temperature. Marked and broad peaks were observed in Figure 4, which resulted from the transition in molecular mobility, representing the *T*_g_ of the polymers. It can be seen from Figure 4a that the maximum peaks are located at 10.2 and 41.6 °C, corresponding to the *T_g_* of PHBV and EA–PPC, respectively. For PHBV/EA–PPC blends with less than 3 parts (wt%) of Sn(Oct)_2_, the curves showed two peaks, in which the peak at the lower temperature corresponded to the *T_g_* of PHBV, and the peak at the higher temperature corresponded to the *T_g_* of EA–PPC. With the increase in the amount of Sn(Oct)_2_, the distance between the two peaks gradually decreased until they merged into one, which was consistent with the DSC results.

### 3.3. SEM Analysis

**Figure 5:**
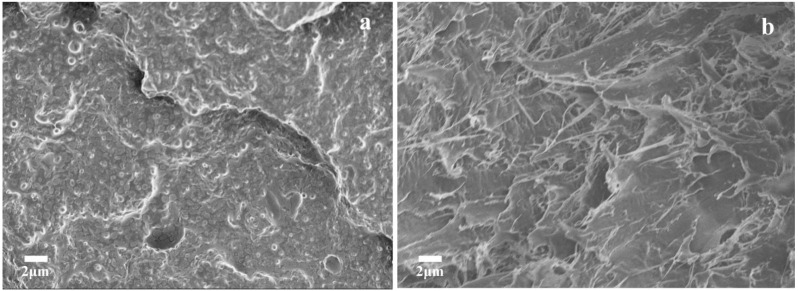
SEM micrographs of tensile-fractured surfaces: (**a**) P70/EP30 and (**b**) P70/EP30/S3 blends.

To further explore the effect of catalytic transesterification on the behavior of tensile fracture during the tensile test, SEM characterization was performed on the tensile-fractured surfaces of P70/EP30 blends without Sn(Oct)_2_ and P70/EP30/S3 blends with 3 parts (wt%) of Sn(Oct)_2_, as shown in Figure 5.

In the absence of Sn(Oct)_2_ (Figure 5a), spherical EA–PPC particles could be distinguished from the smooth brittle failure surface, and there was a clear boundary between the dispersed phase EA–PPC and the continuous phase PHBV matrix, presenting a typical “sea-island” structure. This indicated that the transesterification of PHBV and EA–PPC hardly occurred without Sn(Oct)_2_, leading to poor compatibility, weak interfacial adhesion, and a distinct phase interface between PHBV and EA–PPC. As one can see from Figure 5b, when the Sn(Oct)_2_ content was 3 parts (wt%), the tensile-fractured surface of P70/EP30/S3 blends exhibited a “sea-sea” structure and an obvious ductile fracture. This was because Sn(Oct)_2_ catalyzed the reactive sites between PHBV and EA–PPC, and then the reaction rate of transesterification between the two polymers was improved significantly. The resulting copolymers and oligomers could be used as in situ compatibilizers and plasticizers for the blends, respectively, which increased the free volume among the polymer chains and improved the toughness. The blends softened by EA–PPC can be stretched synchronously with the tensile process, and the plastic deformation spread over the surface of ductile failure. The external stress could be absorbed and transferred effectively before the final fracture, resulting in increased impact strength and elongation at break.

### 3.4. Formation Mechanism of PHBV/EA–PPC Blends

**Figure 6:**
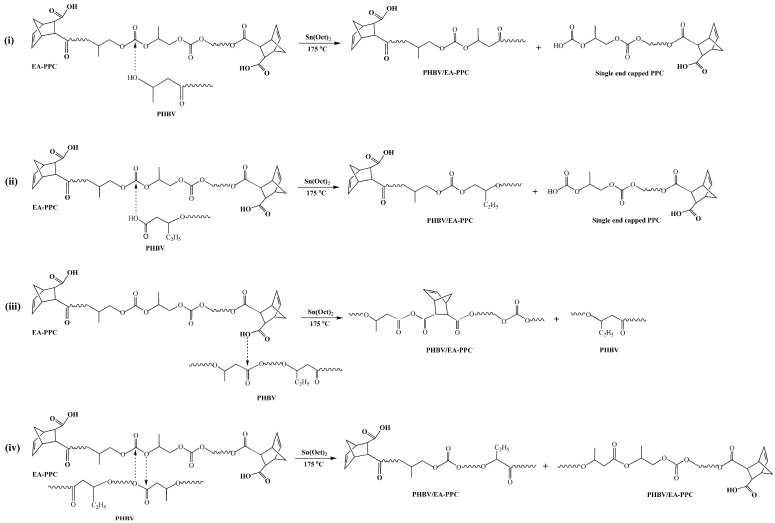
Formation mechanism of PHBV/EA–PPC blends. (**i**) terminal hydroxyl group of PHBV attacked carbonyl carbon of EA–PPC, (**ii**) terminal carboxyl group of PHBV attacked carbonyl carbon of EA–PPC, (**iii**) terminal carboxyl group of EA–PPC attacked carbonyl carbon of PHBV, (**iv**) direct transesterification.

According to the research on the transesterification reaction mechanism in other polymer blends [35,36,37], we speculated that the reaction between PHBV and EA–PPC was a transesterification reaction, as shown in Figure 6. There were mainly four types of transesterification reactions that occurred. The first was that the terminal hydroxyl group of PHBV conducted a nucleophilic attack on the carbonyl carbon of EA–PPC to carry out an alcoholysis reaction under the catalysis of Sn(Oct)_2_. The C-O bond of EA–PPC was broken and the PHBV segment was introduced to generate PHBV/EA–PPC copolymer and PPC segment. The second type was that the terminal carboxyl group of PHBV attacked nucleophilically the carbonyl carbon of EA–PPC, during which an acid hydrolysis reaction occurred. Similarly, the C-O bond was broken to introduce the PHBV segment, generating the PHBV/EA–PPC copolymer segment with different structural units from the first one. Unlike the previous two types of reaction modes, the nucleophilic reagent was EA–PPC instead of PHBV in the third one. In this case, the terminal carboxyl group of EA–PPC launched a nucleophilic attack on the carbonyl carbon of PHBV, which made the C-O bond of PHBV cleave, and then the PPC segment was introduced to the PHBV chain while leaving the remaining PHBV segments. The last way was direct transesterification, also known as the esterolysis reaction [38]. The O of the ester group in PHBV attacked the C of the carbonate group in the PPC chain, and meanwhile, the O of the carbonate group in PPC attacked the C of the ester group in the PHBV chain, forming two new block copolymers at the same time. Carbonate functional groups were less electrophilic than the ester functional groups. Therefore, the third type of reaction was probably the main mechanism by which the transesterification took place. Regardless of the way in which the transesterification occurred, the resulting copolymer can act as an in situ compatibilizer for PHBV and PPC and then improve the compatibility between them.

## 4. Conclusions

PHBV was blended with EA–PPC via melted transesterification under the catalysis of Sn(Oct)_2_ to obtain the PHBV/EA–PPC blends. Effects of Sn(Oct)_2_ dosage on the mechanical properties, thermal properties, and morphology of the blends were investigated. The results demonstrated that EA–PPC can improve effectively the toughness of PHBV and reduce its crystallinity. For the P70/EP30/S3 blend, it exhibited the best toughness. Compared with neat PHBV, the impact strength and elongation at break increased by 59.5% and 93.5 times, respectively, while there was no significant decrease in tensile strength. With the increase in the amount of Sn(Oct)_2_, the difference between the two *T_g_* values decreased until they merged into one, representing the improved compatibility between PHBV and EA–PPC. In addition, the *T_c_*, *T_m_*, and $\chi_{c}$ of the blends decreased with the increase in the amount of Sn(Oct)_2_. It was proved that the resulting copolymer and PHBV (EA–PPC) oligomer produced by the melt transesterification reaction played the role of in situ compatibilizer and plasticizer, respectively.

## Figures and Tables

**Table 1:** Mechanical properties data of PHBV/PPC blends.

Samples	PHBV (wt%)	PPC (wt%)	Sn(Oct)_2_ (wt%)	Tensile Strength (MPa)	Impact Strength (kJ/m^2^)	Elongation at Break (%)
PHBV	100	0	0	39.6	3.7	4.1
PPC	0	100	0	13.8	6.2	483.7
PH80/P20	80	20	0	28.2	4.0	9.8
PH70/P30	70	30	0	26.9	4.3	12.4
PH60/P40	60	40	0	21.7	4.5	13.1
PH80/P20/S1	80	20	1	28.4	4.1	10.3
PH70/P30/S1	70	30	1	27.2	4.5	12.8
PH60/P40/S1	60	40	1	22.1	4.6	13.5

**Table 2:** Data for the mechanical properties of PHBV/EA–PPC blends.

Samples	Tensile Strength (MPa)	Impact Strength (kJ/m^2^)	Elongation at Break (%)
PHBV	39.6	3.7	4.1
EA–PPC	13.2	6.0	458.1
P70/EP30	30.2	4.0	129.6
P70/EP30/S1 ^a^	32.7	4.7	290.3
P70/EP30/S2	36.5	5.6	332.1
P70/EP30/S3	36.8	5.9	387.5
P70/EP30/S4	31.5	4.8	102.7
P70/EP30/S5	29.1	4.3	64.2

^a^ P70/EP30/S1 stands for the blend in which the ratio of PHBV/EA–PPC/Sn(Oct)_2_ was 70:30:1.

**Table 3:** Data for the thermal properties of PHBV/EA–PPC blends.

Samples	*T_c_* (°C)	*T_g_* (°C) from DSC	*T_g_* (°C) from DMA	*T_m_* (°C)	$\chi_{c}\;(\%)$
PHBV	120.6	6.3	9.2	171.7	69.8
EA–PPC	-	34.5	40.3	-	-
P70/EP30	119.3	6.4/34.3	9.8/40.2	171.2	67.1
P70/EP30/S1	114.7	12.3/34.0	13.4/38.3	169.8	48.6
P70/EP30/S2	109.2	23.7/33.9	24.1/38.0	166.6	40.5
P70/EP30/S3	105.2	33.8	37.5	164.3	27.4
P70/EP30/S4	103.1	33.5	-	161.7	26.2

## Sample Availability

Samples of the compounds are not available from the authors.
